# Supplementary material for: Molecular characterization and drug resistance patterns of Mycobacterium tuberculosis complex in extrapulmonary tuberculosis patients in Addis Ababa, Ethiopia
Source: PLoS One. 2020 Dec 7;15(12):e0243493. doi: 10.1371/journal.pone.0243493 (PMC7721190; doi:10.1371/journal.pone.0243493)
Supplement: Attachment — Submitted filename: Response to Reviewers.docx [file pone.0243493.s002.docx]

**Ref: PONE-D-20-17590**

**Molecular Characterization and Drug Resistance Patterns of *Mycobacterium tuberculosis* Complex in Extrapulmonary Tuberculosis Patients in Addis Ababa, Ethiopia**

Dear Editor,

Thank you very much for your informative comments on our manuscript entitled “Molecular Characterization and Drug Resistance Patterns of *Mycobacterium tuberculosis* Complex in Extrapulmonary Tuberculosis Patients in Addis Ababa, Ethiopia”. We have addressed your comments one by one. We also appreciate you for allowing us to revise our manuscript and correct errors in the previous version. We thank the reviewers for their informative comments, and our point-by-point responses to the reviewers’ comments are given below. Also, we would like to inform you that we have used highlights to indicate where we made changes in response to the reviewers’ comments.

**Review Comments to the Author**

Please use the space provided to explain your answers to the questions above. You may also include additional comments for the author, including concerns about dual publication, research ethics, or publication ethics. (Please upload your review as an attachment if it exceeds 20,000 characters)

**Reviewer #1:** Manuscript by Diriba et al reports on the characteristics of M.tuberculosis strains isolated from extrapulmonary TB patients residing in Addis Ababa (Ethiopia). Specifically, phenotypic drug susceptibility patterns and spoligotypes of 151 MTBC strains were analysed and reported. Subject of the study is of interest as Ethiopia still very much remains a white spot in TB world phylogeography while drug resistance patterns of MTBC strains circulating there are still poorly understood especially in extrapulmonary TB isolates.That said, in my opinion, the manuscript has a number of serious problems which need to be fixed before it can be considered for publicatrion.

1. First of all, quality of written English is poor and certain fragments are not fully comprehensible. This applies predominantly to Introduction and Discussion section. Grammar is key problem (passive/active voice; tenses etc). Text should be proof read, ideally by a native speaker, and revised/corrected accordingly.

**Response:** Thank you very much for your informative comments. We have addressed you comments across the manuscript. Errors related to language usage and grammer have revised by native English speaker.

2. Content wise, study design is a key problem. Authors refer to one published and one unpublished study when describing study settings and patient population, but it's far from clear how many specimens were taken from what study, how patients were enrolled and specimens collected (consecutive specimens? convenience sample? individial vs serial isolates? inclusion criteria? etc). Having read the paper authors were referring to (Fantahun et al., PLoS ONE 2019) i realised it included 152 specimens and nearly all the clinical, epidemiological and demographic characteristics were almost the same (current study included 151 specimens), so it's worth expanding and clarifying on whether exactly the same selection of isolates was tested in the current study.

**Response:** Thank you for the pertinent comment. To address this comment we have described the process of sampling and how many isolates were taken from the previously published study and unpulished study as follows

The current study was conducted on clinical isolates of MTBC from two previous EPTB studies conducted in Addis Ababa, Ethiopia. One of the survey data used in the current study was published and the study participants were 152 patients with EPTB (17) while the second survey which is not published was enrolled 778 patients. Sixty eight EPTB presumptive patients were MTBC positive out of 152 (from the first survey), whereas 85 were positive from 778 patients (from second survey). In total, 153(16.5%) MTBCs isolates were obtained from the total of 930 patients from the two previous studies. Since two isolates were not recovered by sub-culturing, 151 MTBC isolates were used in the present study. In both studies, patients were enrolled consecutively from upon the arrival of the participants. In the previous studies from which isolates used in the current study, single sample per patient was collected and tested for TB using culture and Xpert MTB/RIF assay.

3. Depending on representativeness of the study population (which should be clarified as discussed above), all the conclusions and considerations with regards to prevalence of specific drug resistances, spoligotypes, and other characteristics vs other Ethiopian studies and reports from other countries should be adjusted. If this collection was essentially a convenience sample, no firm conclusions regarding prevalence could be made.

**Response:** Thank you for the critical observation. We have retrieved MTBC clinical isolates from the previous two EPTB studies. We believe these two studies were based on statistically determined sample size and the sampling the study participants are represented of their respective study population. This study mainly aimed to document the genotypic and phenotypic charactersitics of the isolates from the two studies. Thus, there are clear difference between previous studies and our study aims.

4. Another problem is hypothesis and principal aim of the study. What was the reason for genotyping and how exactly this type of knowledge can contribute to TB management in Ethiopia especially (as authors rightly state) EPTB is not as transmissible? I suggest to put this study into a broader context of TB/MTBC phylogeography; to this end use of another database (VNTR-MIRUplus) as an alternative or compelentary to SITVIT could be recommended.

**Response:**

Thank you very much for your crtical observation. We have addressed your comment by adding the reasons why gewnotyping method was used and the value of this technique for the TB management in Ethiopia (line 78-80). Since we did not conduct VNTR-MIRUplus due to lack of the technique in our setup, we could not compare our result with the indicated database as complimentary to SITVIT databse. To address this comment we have added a sentence in the limitation part of this study.

5- Other points: author state that "Lymph node TB was the most common site involved..." (Page 9, Line 211-212). However, this will be heavily influenced by inclusion criteria (see above) - if only patients suspected of having Lymph node impairment have been enrolled, than this is not a surprising finding. As I say, defining inclusion criteria will help significantly.

**Response:** We have indicated as "Lymph node TB was the most common site involved..." just to give good insight about the source of the MTBC isolates in detail. We have removed this sentence from the discaussion parts to address your comment.

6- PZA sensitivity: authors rightly state that reproducibility of phenotypic PZA testing is not ideal. It's worth referring to the recent WHO guidelines (eg 2018) where sequencing is recommended as a reference method.

**Response:** Corrected accordingly to the comment. We have indicated in the discussion section as WHO has recently recommends sequencing as a reference method (Line 196-201)

7- Both Background and Discussion sections, as well as fragments of Materials and Methods are far too long and should be shortened. In Introduction. authors should concentrate on their hypothesis and what is known so far; in Discussion messages should be more concise. Pages 10-12 are very difficult to read as they are overloaded with non-relevant information, this should be significantly shortened. In Methods (Page 6) a simple reference to Spoligotyping methodology (eg Kamerbeek et al) will suffice, no need to describe it in details.

**Response:** Thank you for the pertinent comment and suggestion. We have corrected all errors based on your comment.

**Reviewer reports: #2**

**Reviewer #2:** The molecular typing of EPTB isolates (which is often neglected by programme managers) from Ethiopia is appreciated. The samples size is also not very large but acceptable. The findings of M. bovis in about 2% a important finding. However, the study has following concerns and these need to be addressed.

1. The language needs significant improvisation. The Introduction is not very focused on the need of this study. Some of the studies cited in the introduction do not find place in discussion at all, which have clear message and the excerpts should have been cited in discussion as well.

**Response:** Thank you for the informative comments and suggestions. We have revised the introduction to focus to our study aobjective. We have revised the introduction and discussion section based on your comment to clarify and focuse.

2. Like Introduction the discussion is diffuse and lacks a flow. It has a number of repetitions, and irrelevant statement.

**Response:** We removed inrrelevant statements and revised the introduction and discussion sections in detail.

3. The major limitation of the study is that conclusions are based on Spoligotyping. We know that spoligotypic classification lacks discriminatory power and therefore, MIRU-VNTR must have been done. However, considering that this study is coming from Ethiopia, I would not insist on this aspect, but the authors must create a separate para on the limitation of this study, wherein why MIRU-VNTR could not be done, must be mentioned.

**Response:** Thank you very much for the suggestion. We did not conduct advanced molecular tests such as MIRU-VTRN and sequencing due to lack of the setup. To address this comment, we have included an explanation in the limitation part of the revised manuscript (Line 241-244).

Yours, Sincerely

Getu Diriba
